# Drosophila modelling axonal transport in the face of tau pathology

**DOI:** 10.1186/2193-1801-4-S1-L13

**Published:** 2015-06-12

**Authors:** Amrit Mudher

**Affiliations:** University of Southampton, UK

**Keywords:** Microtubule stabilisation, tau, NAP

Alzheimer’s disease (AD) is a devastating neurodegenerative disease causing irreversible cognitive decline in the elderly. There is no disease-modifying therapy for this condition and the mechanisms underpinning neuronal dysfunction and neurodegeneration are unclear. Compromised cytoskeletal integrity within neurons is reported in AD. This is believed to result from loss-of-function of the microtubule-associated protein tau, which becomes hyper-phosphorylated and deposits into neurofibrillary tangles in AD. We have developed a Drosophila model of tauopathy in which abnormal human tau mediates neuronal dysfunction characterised by microtubule destabilisation, axonal transport disruption, synaptic defects and behavioural impairments. Here we show that a microtubule-stabilising drug, NAPVSIPQ (NAP), prevents as well as reverses these phenotypes even after they have become established. Moreover, it does not alter abnormal tau levels indicating that it by-passes toxic tau altogether. Thus, microtubule stabilisation is a disease-modifying therapeutic strategy protecting against tau-mediated neuronal dysfunction, which holds great promise for tauopathies like AD.

